# Research in Integrated Care: The Need for More Emergent, People-Centred Approaches

**DOI:** 10.5334/ijic.5627

**Published:** 2020-10-21

**Authors:** Wilma van der Vlegel-Brouwer, Everard van Kemenade, K. Viktoria Stein, Nick Goodwin, Robin Miller

**Affiliations:** 1 University of Applied Sciences Utrecht, NL; 2 International Journal of Integrated Care, AU

**Keywords:** emergence, paradigms, complexity, people-centred, research methodologies

## Abstract

The International Foundation for Integrated Care (IFIC) recently celebrated its 20^th^ International Conference (ICIC20) through a virtual event that brought together patients and carers, academics, care professionals, NGOs, policy-makers and industry partners from across the global integrated care community [1]. The International Journal for Integrated Care (IJIC) used this opportunity to host a workshop on published research in integrated care, specifically to reflect on the quality of existing scientific enquiry. A lively discussion on the current state of integrated care research concluded that there remained significant shortcomings to current methodologies – for example, in their ability to provide the depth of understanding required to support the knowledge needed to best inform policy and practice, particularly when addressing people-centredness. In part, the debate recognized how the nature of existing research funding, and prevailing attitudes and preferences towards certain research methodologies, were partly to blame (as has been noted by IJIC previously [2,3]). The workshop debated how research and researchers must change their focus in order to better contribute to the tenet of people-centred integrated care.

## Quality paradigms in integrated care

The workshop – led by Wilma van der Vlegel-Brouwer and Everard van Kemenade - began with a reflection on recent research by them that had identified four quality paradigms for integrated care: the Empirical, Reflective, Reference, and Emergence paradigms [4,5].

In the *empirical paradigm* the main objective is to measure reality and guide knowledge production to contribute to evidence-based medicine. Research is mainly based on positivism and aims to explain, predict and control. The *reference paradigm* adds to the improvement of client care by using models, frameworks, protocols or guidelines to develop and evaluate care. Research in this paradigm, often based on constructivism or interpretivism, aims to understand and reconstruct. In the *reflective paradigm* the professional or group of professionals is the expert who reflects on the quality of care. Research in this paradigm, based on subjectivism, aims to critique and transform practice. In the *emergence paradigm*, the latest paradigm, a collective of stakeholders, including patients and/or citizens, explore and co-create new solutions. This is underpinned by the research philosophy of pragmatism or participatory research. It aims to inform our understanding of the dynamic interactions and lead to novel practice which respond to the real world context of local levels.

The empirical and reference paradigms fit best in circumstances that are certain or can be planned; the reflective and emergence paradigms fit best in circumstances which are uncertain and cannot be planned. Therefore the science of integrated care would greatly benefit from ‘epistemic fluency’ (i.e. applying knowledge from all four paradigms). As a result, Van Kemenade & Van der Vlegel-Brouwer (2019) proposed a new overarching definition of integrated care, which acknowledges all four paradigms: *Integrated care is the process of help, care and service, managed and coordinated by interconnected highly competent professionals, who by their synergy – together with the client and his family as partners – find solutions and create impact, continuously adapting to the context and circumstances.*

Based on these findings, van Kemenade and van der Vlegel-Brouwer presented results from a new study that aimed to explore the use of quality paradigms in integrated care research. By assessing all abstracts in IJIC published between January 2015 and December 2019 (n = 258) every article was placed in one of the four quality paradigms. The presence of each paradigm was studied in different contexts, looking at the countries of origin, the domain of impact in research, policy or practice and the role of the patient in the research. Discrepancies were resolved by reading the article in full and discussion between the researchers. Summarising the results, the reference paradigm appeared to be most prevalent since 147 (57.6%) studies were placed in this paradigm. In addition, 55 (21.6%) of the studies were placed in the empirical paradigm and 45 (17.6%) of the studies were placed in the reflective paradigm. Only 8 (3.1%) of the studies were placed in the emergence paradigm. This might implicate that values like accountability and accuracy (empirical paradigm) and success and improvement (reference paradigm) prevail over professionalism and wisdom (reflective paradigm) and flexibility and willingness to change (emergence paradigm). An active role of the patient, whether consultative or collaborative, was found in less than 20 percent of all the studies [6].

## Implications for the future of integrated care research

During the workshop, surprise was expressed that within the reviewed articles, and consequently across integrated care research itself, the reflective and emergence paradigms seem to be underrepresented. This is noteworthy since these two paradigms fit best if one wants to conduct research in contexts with complexity and uncertainty – i.e. the reality of integrated care design, implementation and evaluation [2,3,7,8]. It is therefore surprising that the paradigmatic underpinnings in the articles published in IJIC from 2015 to 2019 reflect these paradigms (and therefore related contexts) only marginally.

The lack of attention to using complexity theory and associated methodologies to investigate integrated care was recognized as a problem by workshop participants. It was suggested that researchers often prefer to use the reference paradigm framework because the prerequisites for research projects include detailed planning. The formulation of hypotheses and expected outcomes drive such an approach. Very often, researchers are not granted the flexibility to *not* religiously plan or to allow for emergent and evolving results, or indeed recognize that interventions necessarily must vary to suit the unique contexts in which they are implemented. Education in research methods also focuses on empirical and reference methodological frameworks. It therefore favours static, predictable and planned approaches to research and implementation. All workshop participants recognized this as a significant knowledge deficit and so called for a drastic shift in how research institutions, researchers’ attitudes and research funders should promote and accommodate alternative approaches that are likely to better capture and reflect the complexity and emergent character of integrated care.

## People-centred care needs people-centred research

The other hot topic of the discussion was the importance of including people’s perspective in research, but the distinct lack thereof in actual research. Integrated care is by definition person-centred and integrated care strategies should be based on co-creation with patients or citizens in ways that involve, engage and empower them. How do our research approaches capture this? Similar to integrated care design and implementation, where approaches might profess to be person-centred but in reality are not, there is also a chasm between theory and practice. So, as with integrated care practice, there is a need for co-creation frameworks for research. As researchers, it would be hypocritical to criticise care professionals for not involving patients, families and communities more effectively in their care while at the same time failing to actively involve them in our research.

Van Kemenade & Van der Vlegel-Brouwer’s presentation clearly unveiled a consistent bias that exists in both research as well as in practice to the involvement and engagement of patients, carers, families and wider community stakeholders. This is to the detriment of everyone and, especially in studies on integrated care, we seem to be missing the point of co-production and not practicing what we preach. Research needs to learn to redistribute power back towards people and gravitate away from a system in which professionals and researchers know best. Leadership in academic and funding institutions should support this movement towards co-production and adjust project requirements accordingly. Perhaps more fundamentally, integrated care needs to depart from the traditional positivist health mindset that continues to, dominate our thinking. Over the past 20 years, our understanding of integrated care has become much more person- and community-centred as well as multi-sectoral. It is time researchers took a leaf out of other books – for example, of working with people with disabilities, which is far ahead in the integrated care field in terms of co-production of research and implementation [e.g. 9,10].

## Recommendations for integrated care researchers

A final reflection and challenge from the workshop was that articles published in IJIC should be much more clear on their perspective, about the aim of their research and about the context wherein the research was done and how this has influenced the methodology of the study. More attention should also be paid to co-creation with people in research itself, implying that IJIC and its reviewers should become more demanding of methodologies to demonstrate inclusive practices. This may be a transitional process, not least due to the different research traditions around the world and their evolutionary stage in embracing new research practices. Nonetheless, if this retrospective study is to be repeated in another ten years’ time, we would hope to see more work that embraces the reflective and emergent paradigms and demonstrates inclusion of people and communities in their design and implementation.
